# Alpine Skiing Activity Recognition Using Smartphone’s IMUs

**DOI:** 10.3390/s22155922

**Published:** 2022-08-08

**Authors:** Behrooz Azadi, Michael Haslgrübler, Bernhard Anzengruber-Tanase, Stefan Grünberger, Alois Ferscha

**Affiliations:** 1 Pro2Future GmbH, Altenberger Strasse 69, 4040 Linz, Austria; 2 Institute of Pervasive Computing, Johannes Kepler University, Altenberger Straße 69, 4040 Linz, Austria

**Keywords:** human activity recognition, alpine skiing, unsupervised learning, inertial measurement units

## Abstract

Many studies on alpine skiing are limited to a few gates or collected data in controlled conditions. In contrast, it is more functional to have a sensor setup and a fast algorithm that can work in any situation, collect data, and distinguish alpine skiing activities for further analysis. This study aims to detect alpine skiing activities via smartphone inertial measurement units (IMU) in an unsupervised manner that is feasible for daily use. Data of full skiing sessions from novice to expert skiers were collected in varied conditions using smartphone IMU. The recorded data is preprocessed and analyzed using unsupervised algorithms to distinguish skiing activities from the other possible activities during a day of skiing. We employed a windowing strategy to extract features from different combinations of window size and sliding rate. To reduce the dimensionality of extracted features, we used Principal Component Analysis. Three unsupervised techniques were examined and compared: KMeans, Ward’s methods, and Gaussian Mixture Model. The results show that unsupervised learning can detect alpine skiing activities accurately independent of skiers’ skill level in any condition. Among the studied methods and settings, the best model had 99.25% accuracy.

## 1. Introduction

Alpine skiing is an interesting, competitive, and complex sports activity that has been a part of the Winter Olympics since the first event. Most studies in this area focused on performance analysis where scholars analyzed the biomechanics of skiers to find the main factors affecting alpine skiing performance such as turning techniques, aerodynamic drag, ground reaction force, or turn radius [1,2]. While skiers attempt to optimize these factors to enhance their performance, the risk of injuries will increase [3]. Therefore, scholars developed an interest in related factors to injury risk [4]. In [5], authors studied the effect of ski sidecut on turning mechanics in the context of injury prevention. This motivated scholars to examine turns in detail as the fundamental part of alpine skiing [4] and develop methods to detect turns [6,7].

The mentioned studies utilized several different sensors ranging from video to wearable sensors. Additionally, most of the analyses have been performed under controlled conditions in laboratories or through a limited number of gates. One potential solution to expand these studies is the use of inertial measurement units (IMU) to detect entire activities during a whole day of skiing where a skier performs several skiing techniques. Therefore, it will be possible to analyze each activity in detail.

In recent years, there has been an increasing interest in research on human activity recognition (HAR) due to advances in wearable and visual sensors [8]. The goal of HAR is to classify incoming signals from human motions into different categories of activities such as human daily activities. Despite the wealth of analysis on human daily activities, the application of HAR to alpine skiing activities has not been researched extensively [9]. Additionally, many scholars have employed various sensors from visual to commercial IMUs that are not suitable for daily use.

In [10], scholars investigate finding the place on the skier’s body to attach an inertial sensor so that it is possible to collect the most informative signals. They concluded that the pelvis is the best place to locate a sensor. Although, there the other parts of the body which could be alternatives due to the similar results in their analysis. In [7], the authors developed an algorithm to detect the starting point of a turn, which can be utilized for regular use. In another study on turn detection [11], the use of gyroscopes was examined. The data collection for both of these studies was carried out under controlled conditions and still needs validation in the wild. These studies show that turn behavior is well represented in IMU signals which is a key to distinguishing alpine skiing activities. In [12] they classified four popular turning styles, snowplow, snowplow-steering, drifting, and carving, using a global navigation satellite system (GNSS) and IMU. They analyzed a dataset of 2000 turns from 20 advanced skiers. In another study, Han et al. employed a motion sensor and piezo transducer to collect data from subjects. Then, they analyzed the collected data in a supervised manner to predict the status of a skier during winter sports such as alpine skiing and snowboarding [13]. In [14] three IMU sensors were attached to the skier’s chest and skis to collect data from several skiers on one slope. This dataset is analyzed using two long short-term memory (LSTM) networks for skiing activity recognition to detect left/right turn, left/right leg lift, ski orientation, and body position. Although the LSTM classification results show high accuracy, the model needs more validation in different locations under varied conditions. Additionally, they did not report the skill level of subjects in their study.

Our ultimate goal is to provide recreational alpine skiers with performance analysis insights about their skiing. However, in this study, the primary goal is to have an unobtrusive sensor setup feasible for daily use along with an algorithm that is able to distinguish between skiing and not skiing. Today, access to smartphones equipped with IMUs offers the possibility to detect different sorts of activities on the phone [15]. In this work, we investigate the application of unsupervised machine learning in alpine skiing activity recognition of recreational alpine skiers using smartphone IMU. Often, HAR has been formulated as a supervised task [15,16]. Here, we study the use of unsupervised learning in distinguishing skiing activities from other activities. We prefer unsupervised learning over supervised learning since it is not dependent on large labeled samples [17], which is not easy to gather in the case of alpine skiing. Additionally, in this study, we only need to find the beginning and the end of skiing activities, and we will not differentiate skiing techniques from each other. Moreover, depending on the level of expertise, every subject has a different skiing style, which may affect the supervised learning process negatively due to varying patterns generated by each skier. Finally, employing unsupervised learning increases scalability since we can add more data without any concern about labeling, which eases adding more subjects to the project without prior knowledge about the data labeling.

In the rest of the paper, we give an explanation of data collection in detail. Then, we go through data preprocessing, including orientation tracking, filtering, and feature engineering. The results section compares the results from different settings and algorithms. Finally, we summarize the experiment and present future works.

## 2. Materials and Methods

### 2.1. Data Collection

**Figure 1:**
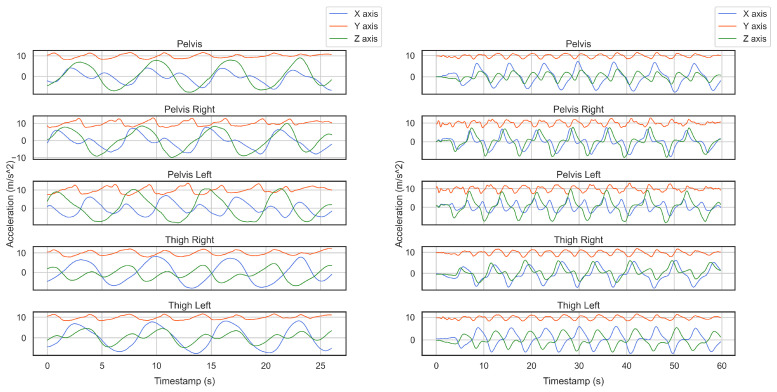
We located two smartphones on the right and left pelvis and three Xsense sensors at the pelvis, right thigh, and left thigh to record alpine skiing activities. As diagrams imply, all the sensors recorded similar periodic patterns of skiing.

Overall, we conducted six data collection sessions, see Table 1. In the first two recordings, we employed fourteen Xsens sensors to follow the research done by [10] where they concluded that sensors attached to the pelvis, right and left thigh generate highly similar patterns, and the pelvis is the best place to locate a sensor. As in our study, the aim is to have only one smartphone in the skier’s pocket, which could be at the right/left thigh or right/left pelvis, so we attached two smartphones to the right and left pelvis and two Xsens sensors at the right and left thigh to examine their similarities in captured patterns. Our very first result showed that the incoming data from these smartphones had characteristics in common with the sensor placed at the pelvis. In Figure 1, one can see the similarity between smartphones and Xsens sensors from two different activities. Therefore, in the rest of the study, we only use one smartphone placed on the right thigh. The smartphone’s IMU returns acceleration and angular velocity signals with a frequency of 500 Hz.

We asked subjects to ski several times with different techniques. These techniques are chosen by an experienced alpine skiing instructor based on the Austrian Ski Instruction Teaching Plan that consists of four stages: Green, Blue, Red, and Black, from start to perfection. These techniques are as follows: Parallel Basic-Long (red), Parallel Basic-Short (red), Parallel Dynamic-Long (red), Parallel Dynamic-Short (red), Race Carving-Long (black), and Race Carving-Short (black). Each subject takes a lift to go up the piste, and from there, skis a bit to reach the starting point, and then starts driving for at least 30 s to finish an activity at the lift. Therefore, each session includes different sorts of activities other than alpine skiing, e.g., getting on the lift. In this study, we tried to consider a high level of complexity in data collection. Therefore, data have been recorded in varying seasons at several places on different slopes, see Table 1. Additionally, we collected data from skiers with various abilities to validate our approach to recognizing alpine skiing activities because skiers with varying levels of expertise ski differently.

In the first recording session, two advanced and two intermediate subjects performed three techniques each. Then, a novice user was asked to ski five different alpine skiing styles in the second session. In the third data collection, an expert user skied all the alpine skiing styles three times on different slopes. He performed all six skiing styles and skied some of the techniques more than once. During the fourth data gathering, the other expert performed all six skiing techniques. Lastly, at Ramsau, we recorded data at longer sessions, in terms of time duration and the number of activities, from four subjects with different capabilities, see Table 1. Since these sessions are long, they are comparable to a day of skiing. In three of these sessions, three of the subjects skied for about four hours and the other one for more than one hour.

For ease of data collection, we developed a mobile app so that any user will be able to record their data simply on their smartphones. Thus, self-recorded in the Table 1 means that the user recorded a skiing session on their smartphone via our application. In the fourth session, we trained users on using the application and started the application on their smartphones. Therefore, we consider this session partially self-recorded. Finally, the term Not self-recorded means that the skier only carried the smartphone and was not involved in attaching the phone or controlling data recording processes.

### 2.2. Experimental Design

**Figure 2:**
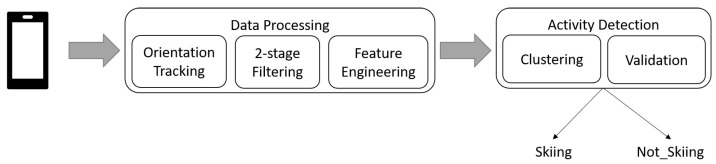
The schematic architecture of the proposed algorithm setup. This pipeline consists of two main modules: data processing to preprocess incoming signals and activity detection to classify preprocessed data into two categories of skiing and not skiing. The smartphone is only used for data collection.

For our experiment, we implemented a pipeline to analyze incoming data from each user, see Figure 2. Our pipeline begins with orientation tracking to transform acceleration signals from the body frame to the world reference. Next, it filters the transformed signals through a two-stage filtering approach and extracts a set of features on the filtered signals based on a windowing strategy. Finally, it clusters the extracted features into two classes Skiing and Not_Skiing. At validation, the pipeline drops activities that are not long enough (shorter than 30 s) which means that any alpine skiing activity must be at least 30 s. This threshold avoids mixing up the other short semi-skiing actions with alpine skiing activities. Additionally, we ensure that in activities with long turns e.g., Race Carving Long, at least two turns are performed within 30 s. In the following sections, we explain each of these modules in detail.

#### 2.2.1. Orientation Tracking

In the first step, our analysis starts with orientation tracking, where we fuse the acceleration and angular velocity signals to transform the acceleration from the body frame to the world reference frame. This approach helps in isolating gravity on one axis (here Y-axis) and proper acceleration on the other two axes. To have such an IMU-based motion tracking, we followed the orientation tracking with quaternion developed in [18]: 3-DOF orientation tracking with IMUs where they adopted the math and notation from [19] and also Chapters 9.1 and 9.2 of [20].

**Figure 3:**
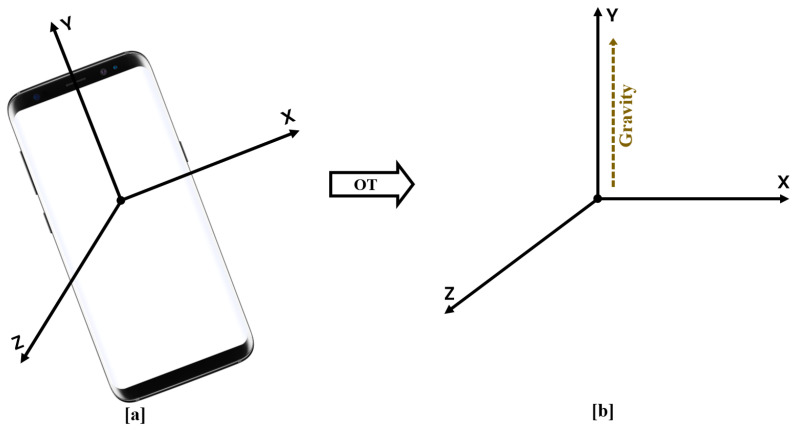
Orientation transformation (OT) from the body frame to the world reference frame. (**a**). Smartphone in an arbitrary orientation. (**b**). Fixed reference system where the Y axis is aligned with the gravity vector.

At each time step, we compute a rotation quaternion using the complementary filter to correct tilt error. This rotation quaternion will correct tilt error by rotating accelerometer measurements from the body frame to the world frame. In the world frame, we will have a fixed reference system, wherein gravity always points up along the y-axis, see Figure 3. Hence, by rotation, we can project the gravity on one axis and the proper acceleration on the other two axes. We apply a complementary filter that contains a low and a high pass filter to fuse the gyroscope and accelerometer to have a better orientation estimation. The accelerometer measurements are reliable in the long term. Therefore, a low pass filter can correct errors in acceleration values and removes high-frequency noise. On the other hand, the gyroscope works accurately in the short term and drifts in the long run. So, a high-pass filter can integrate gyroscope measurements to remove drift.

**Figure 4:**
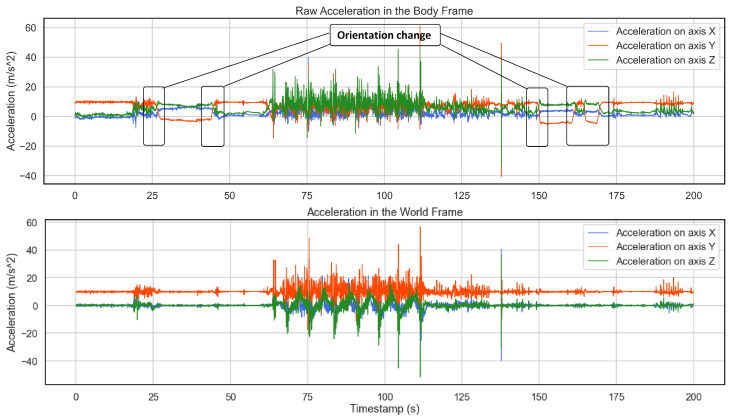
In the raw acceleration plot on the top, the boxes show several changes in the orientation that are corrected in the world frame at the bottom. This diagram also implies that any orientation change in the body frame causes different axes to measure gravity. While in the world frame, gravity is projected on the y-axis.

During a full session of skiing, from time to time, users may check their phone and put it into the pocket in another orientation, any change in the axes could be problematic due to having a different feature for the same axis in the feature space. As a result of orientation tracking, we are not any longer concerned about changes in orientation while recording. Figure 4 shows an example of orientation change during a session where the annotated changes in the body frame are fixed in the world frame. As one can see, in the world frame the gravity is projected on the y-axis.

#### 2.2.2. 2-Stage Filtering

**Figure 5:**
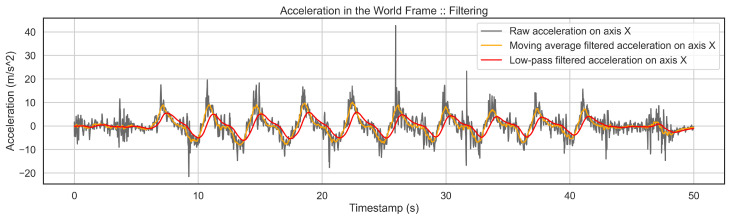
This figure shows a part of the signal before and after filtering. First, a moving average is applied to raw signals to keep the permanent patterns. Then, the 1st filtered signal is passed to a low-pass filter to achieve the 2nd filtered signal. One can see that the two-stage filtering has nicely removed all the high-frequency noise, and we get a clear pattern of the activity.

Filtering of Data is necessary due to the shaking of the smartphone in the user’s pocket and skiing conditions such as the quality of snow. Therefore, the accelerometer and gyroscope channels from the world frame are filtered twice to reduce all the mentioned effects to a minimum level. First, a moving average filter is applied to keep permanent patterns in the entire signal. Second, a low pass filter keeps a clear and smooth pattern of the activities and drops noise nicely. Figure 5 shows the effect of filtering on a signal where the high-frequency noise is removed, and a smooth pattern of activity is kept.

#### 2.2.3. Feature Engineering

In the feature engineering step, we extract a set of features from each signal required by machine learning algorithms. We follow a windowing strategy to divide signals into smaller segments and extract several features so that, from each window, various features will be calculated. These features have been studied and used in the literature for HAR purposes [21,22,23]. The list of features is as follows:

- mean, standard deviation, root mean square, minimum, maximum, median, variance, median absolute deviation, the energy of the window and its auto-correlation
- mean crossing, 50 percent crossing, 25 percent crossing, 75 percent crossing of the window and its auto-correlation
- mean, the median of Power Spectrum of the window
- SMA: Signal Magnitude Area

**Figure 6:**
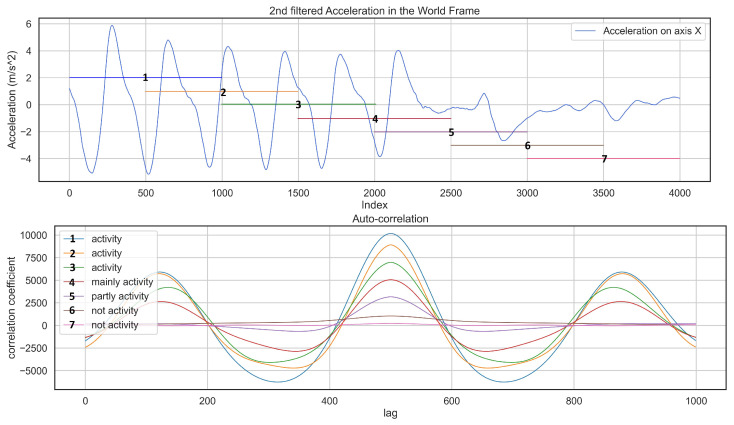
The plot on the top shows a part of the signal which contains an activity. The one at the bottom shows an auto-correlation result based on a window of 8 s moving from left to right. Each arrow shows a window of 10 s with color with respect to auto-correlation.

The auto-correlation technique is useful in finding repetitive patterns in a signal since it keeps the same properties as the original signal. Figure 6 represents how auto-correlation detects the presence of periodic patterns inside a signal where the windows containing a skiing activity have a higher correlation coefficient.

After extracting features, we normalize these values via min-max scaling to the range of [−1, 1]. This normalization is necessary for unsupervised learning since the majority of clustering methods, as well as Principal Component Analysis (PCA), will not work without normalization [24]. The normalized data set of features is called the normalized feature set (NFS). Then, we apply PCA to the NFS data set to reduce the dimensionality and get another data set which we name PCA. Having two feature sets (FST) enables us to train more models and find the best solution.

#### 2.2.4. Clustering

We consider any alpine skiing activity as an active session to detect for further analysis. Therefore, the task here is to divide the input signal into two clusters: Skiing and Not-Skiing. Since we have only two classes, clustering algorithms will be initialized to detect two clusters of activities. In this experiment, we employ three different unsupervised learning algorithms: Gaussian Mixture Models (GMM), KMeans, and hierarchical clustering Ward’s method which is widely known and studied by other scholars [23,25,26]. In combination with two datasets from feature extraction (NFS and PCA), we will have six different models as the output of cluster analysis.

#### 2.2.5. Validation

To evaluate our results, we refer to the accuracy of each model and compare it with a baseline. Here, the baseline is considered a model which predicts everything as Not Skiing since this class is the majority class. In this way, we better see how efficiently each model improves the baseline. The accuracy is calculated as a fraction of correctly predicted values over the number of samples. Moreover, having applied unsupervised methods, we use two unsupervised metrics to assess the goodness of clustering: Normalized Mutual Information (NMI) and Adjusted Rand Index (ARI). Both NMI and ARI are external measures that need the ground truth. They score 0 for random labeling and 1 for perfectly complete labeling (negative values are invalid).

ARI [27] is a measure of similarity between two data clustering. The ARI is calculated as [28]:

(1) $$\mathrm{ARI}=\frac{\mathrm{RI}-E[\mathrm{RI}]}{\max(\mathrm{RI})-E[\mathrm{RI}]}$$

where E[RI] is the Expected Rand Index and Rand Index is given by:

(2) $$\mathrm{RI}=\frac{a+b}{C_{2}^{n_{\mathrm{samples}}}}$$

Here, C is considered the ground truth. If we assume K is the clustering result, a is the number of pairs in the same cluster in C and K, and b shows the number of pairs in the different clusters in C and K. And $C_{2}^{n_{\mathrm{samples}}}$ shows the total amount of possible pairs in this dataset.

NMI is computed as [26]:

(3) $$\mathrm{NMI}=\frac{\sum_{i=1}^{r}\sum_{j=1}^{s}n_{i,j}\log(\frac{n\cdot n_{i,j}}{n_{i}\cdot n_{j}})}{\sqrt{\sum_{i=1}^{r}n_{\mathrm{ilog}}\frac{n_{i}}{N}\sum_{j=1}^{s}n_{\mathrm{jlog}}\frac{n_{j}}{N}}}$$

Here, r is the number of true clusters, s is the number of assigned clusters, $n_{i,j}$ is the number of samples in both cluster i and a cluster assignment j, $n_{i}$ is the number of samples in cluster i, $n_{j}$ is the number of samples in assigned cluster j, and N is the total number of samples.

### 2.3. Data Analysis

The window size and sliding rate selection have been discussed a lot in the HAR domain [21,23]. Choosing the right window size and sliding rate plays a critical role in the feature extraction to ensure that all the patterns are covered throughout an incoming signal. Additionally, we must choose the proper algorithm for our analysis that works accurately and fast. Moreover, we have two data sets as the output of the feature selection phase, which in combination with three clustering methods give six different models. In this way, we must find the best setting for the model, feature selection, window size, and sliding rate.

Activities shorter than 30 s will be dropped since we assume that any alpine skiing activity at least lasts 30 s, and activities smaller than this could be similar to activities of interest. To evaluate our selection, we consider the average of metrics (NMI, ARI, and accuracy) and their standard deviation to ensure the algorithm works stable independently of different settings (The provided values in the result section are averaged). The other evaluation metric is the number of detected activities. Each model needs to find the correct number of activities to ensure that the chosen model does not over-fit in the cases where a detected activity is similar to activities of interest.

In the first phase of our analysis, we start with data sets from the first four recording sessions (Table 1) where we have enough complexity of data considering varied skill levels, seasons, and locations. In the rest of this paper, We refer to a setting as any combination of algorithms, datasets, window sizes, and sliding rate. To choose the best combination of window size and sliding rate, we generate features on windows of [3,4,5,…,10] seconds and [0,20,50,80] percent overlap. Through such a tuning search, we can make sure of finding optimal values. To find the best setting, we compare different combinations not only through clustering metrics but also by using their time consumption. Since feature engineering on shorter window sizes takes longer and generates more samples, this directly affects the overall response time.

In the second phase of the analysis, we carry out the same experiment as the first phase on the data sets recorded at Ramsau. The main reason we executed the second phase of the analysis is that the setting in the Ramsau recording is different from the other recordings, see Table 1. In Ramsau recording, all the users recorded their activities on their smartphones, while we employed our smartphone in the first four data gathering sessions. Additionally, we asked them to ski all the techniques focused and unfocused. So, we collect various patterns of activities if they are performed differently. Moreover, these sessions are long enough to be compared with a day of skiing. Therefore, Ramsau recording is more similar to the real skiing scenario.

## 3. Results

### 3.1. Analysis Result: Phase One

In our analysis, first, we find which algorithm works the best independent of the other settings. Results show that KMeans on data set NFS has the highest scores on all the metrics (Accuracy, NMI, and ARI). The Table 2 shows the result of algorithm selection where we can see that Kmeans_NFS works slightly better than the others.

In the second step, we examined window size and sliding rate to see which one gives the best result despite the other settings and skiers. Since there are 32 combinations of window sizes and sliding rates, only the best five are shown in Table 3.

Considering findings from Table 2 and Table 3, the best setting is KMeans as an unsupervised method, NFS as a feature set, and a window size of 6 s with a 100% sliding rate (which means no overlap). To check this further, we compare this setting with the other possible settings. Table 4 shows the five best results, no matter who skies, to have a comparison between our selected setting with the others.

Based on the above analysis, the best setting is a window size of 6-s with a 50% sliding rate for feature extraction and applying GMM on the NFS feature set to detect activities. Although, the other settings in the Table 4 can be an option due to their similar performance. In the next phase, we examine this further to see whether this setting works the best for the other datasets or not.

### 3.2. Analysis Result: Phase Two

As before, we examine the algorithms’ performance independent of the other settings that show Kmeans_PCA has the best performance with average values of 98.94% accuracy, NMI 0.83, and ARI 0.92. In the next step, we check different combinations of window size and sliding rate that indicates window size of 8 s with 100% sliding rate performs the best with 97.5% accuracy, NMI 0.77, and ARI 0.86. Finally, we analyze all the sessions from the Ramsau recording using the chosen setting from the first two steps to find the best setting Table 5. Based on results from the first two steps, the chosen setting should be KMeans_PCA with a window size of 8 s and 100% sliding rate, but it does not show up in Table 5. Scores for this setting are as follows: 99.12% accuracy with NMI 0.85 and ARI 0.93 which is comparable to the best score. This means that we have a handful of models with acceptable performance.

Comparing the last tables of analysis phases one and two, we see that none of the settings shows up in both. As the data collection condition in the Ramsau recording is more realistic, we rely on the results from phase two. Additionally, to choose the most efficient model and setting, we look at their time consumption at different stages. Our analysis shows that applying any of the algorithms on the NFS dataset takes longer than PCA. For example, the average time consumption of all the windowing strategies for one of the sessions recorded at Ramsau is 0.12 s for KMeans_PCA and 0.5 s for KMeans_NFS. Additionally, we are more interested in choosing windowing strategies with longer window lengths and less overlap so that feature engineering would be faster. To sum up, considering the results from Table 4 and Table 5, our final selection is model KMeans_PCA with a window size of 8 s and sliding rate of 50%.

## 4. Discussion

The goal of the study was to detect alpine skiing activities via smartphone IMU in an unsupervised manner that is feasible for daily use. Our result shows that by locating a smartphone IMU in the skier’s pocket on the right side, it is possible to record informative signals to recognize alpine skiing activities using unsupervised learning.

Even though orientation tracking works pretty well in isolating gravity, there is still an issue with proper acceleration decomposition. This problem is referred to as rotation about the gravity vector (in our study Y-axis in the world frame). One possible solution to this obstacle is Yaw correction via employing the magnetometer. This enhancement is especially essential for more analysis where we take a closer look into each activity, classify them in different techniques, and avoid drift in speed estimation.

**Figure 7:**
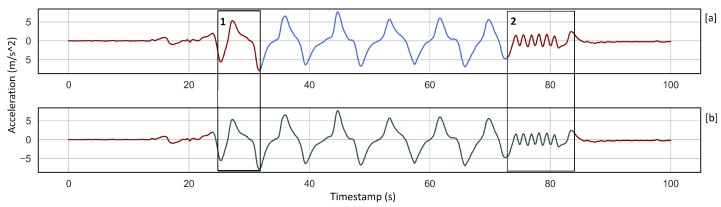
The plot (**a**) shows the ground truth, and diagram (**b**) shows detected activity using KMeans_PCA. Box 1 depicts one cycle of the activity that is not labeled while it is a repeated pattern and recognized by KMeans_PCA. Box 2 illustrates the last part of the detected activity is different from the rest, which is overfitting. This part is an example of semi-skiing activity.

We applied unsupervised learning in our analysis because it gives the possibility to start our study with a small dataset of alpine skiing activities and increase the number of samples incrementally without more training. In contrast, supervised learning needs a large data set of labeled skiing activities which are not easy to collect. This means that we do not use the labeled data in the learning process. However, it is necessary for evaluation. This implies that the quality of data labeling has a direct impact on the assessment of each method, Figure 7a, where a part of the activity is not labeled but is detected. In addition, we do not distinguish different alpine skiing techniques from each other, so we only need to find the beginning and the end of activities. Although the results show that our approach recognizes skiing activities from the rest of the activities with acceptable accuracy, there is still room for improvement.

If we take another look at the detected skiing activities, it is clear that the beginning and end of each skiing activity include some semi-skiing activities, Figure 7b, which we should avoid to have an accurate detection pipeline. This issue is the effect of using fixed-size windows where a window covers the majority of activity and some semi-skiing activity. See similarities between a window of “mainly activity” and “activity” in Figure 6. The other benefit of unsupervised learning is that we can automatically label recorded skiing activities from different recreational skiers into Skiing and Not-Skiing and then classify them in different skiing styles as future work. Additionally, using our mobile application, any skier can easily record their data. This unsupervised approach combined with the mobile application helps considerably in saving time and ease of data collection.

**Figure 8:**
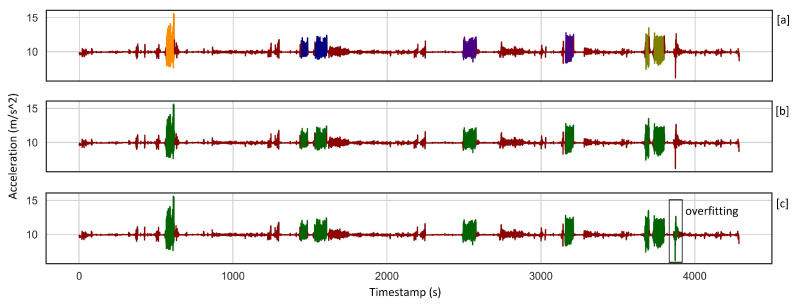
(**a**). Ground truth consists of seven alpine skiing activities, where different skiing styles are shown in different colors. (**b**). Our chosen model detected all the seven activities with 99.17% accuracy, where the green color indicates skiing class. (**c**). GMM_PCA recognized eight activities with an accuracy of 98.12%. One can see the overfitting at the end of the signal, which affects clustering metrics significantly, see Table 6.

Some of the sessions are very long, which causes high accuracy even when some parts of the activities are not detected. In such cases, getting a very high accuracy value does not show that the algorithm works perfectly, while clustering metrics are more descriptive and show these differences. For example, in a session of more than one hour where there are seven skiing activities, our chosen model finds all the seven activities with an accuracy of 99.17, and GMM_PCA recognizes 8 activities with an accuracy of 98.12. While there is no significant difference between these accuracies, their clustering metrics vary considerably. Figure 8 and Table 6 explain these consequences more and show how overfitting affects clustering metrics. So, when the number of samples increases, NMI and ARI are more reliable for evaluation since, as metrics of the goodness of clustering, any overfitting and underfitting affect them negatively.

**Figure 9:**
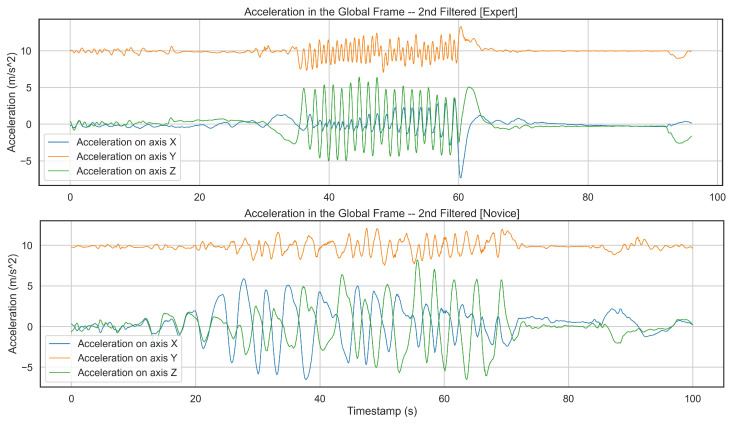
Comparing generated patterns from an expert and a novice skier on different axes of an accelerometer demonstrates that the expert subject skis faster and more consistently as the user finishes the activity in a shorter time than the novice and keeps repeating similar patterns. On the other hand, the novice skier cannot produce such a pattern the whole way.

In our experiment, we tried to consider the highest complexity in the data collection. However, there is still a lack of female skiers. In the future, the proposed algorithm must be evaluated and validated through female skiers with various capabilities, so we ensure that this analysis can detect all the alpine skiing activities independent of users and their physical features. Additionally, we have only two novice skiers. Since novice skiers generate different patterns than more advanced skiers, this approach needs to be examined with more beginner skiers to confirm that it works similarly for skiers with any skill level. Moreover, our chosen model only detects three activities out of five from one of the novice skiers. Figure 9 shows a comparison between two subjects, one expert and the other novice skier. As the figure implies the expert skier performs the skiing activity faster while generating a consistent pattern.

There are three points in the pipeline which are time-consuming and affect the response time. First of all, orientation tracking has to be applied to the entire input signal and is dependent on the number of samples. So, the length and frequency of the input will influence time consumption at this step. One uncomplicated solution for this issue is to sub-sample the input signal to 50 Hz, which is concluded to have enough information for high-frequency activities [29]. Second, feature extraction is heavily dependent on the windowing strategy. Scholars concluded a window size between [2, 3, 4, 5] seconds is ideal for HAR applications [30,31] which are mainly low-speed activities. But, our analysis shows that short-size windows generate a higher number of samples in the feature space than larger window sizes, which takes longer as an issue of time consumption. Here, one solution is assigning larger window sizes where there is no significant difference in the accuracy. Finally, clustering methods are heavily dependent on the number of samples. As it is studied in [32], KMeans works better on larger datasets. Therefore, it can always be an option where input is a large set of features.

## 5. Conclusions

In summary, we provide an easy-to-use sensor setup for data collection so that every skier with any level of mastery can record their skiing data with a smartphone. Then, we examined unsupervised learning to see whether it can distinguish skiing activities from the other activities during one session of alpine skiing. Additionally, we explored different combinations of window size and a sliding rate for feature engineering to ensure we extract the most relevant features. Our result shows that this approach can detect active sessions of alpine skiing for further analysis. Therefore, our pipeline offers an automatic data collection and pre-labeling of alpine skiing signals, which can be used together with other sensors.

## Figures and Tables

**Table 1:** Data collection has taken place in varied locations and conditions, including snow quality and slopes. Subjects in this study have different capabilities ranging from novice to expert, and each performed from three to six different skiing styles. On the first three recordings, data collection has been done using our smartphones (Galaxy S9). In the other two recordings, data collection is done through our application on the subject’s smartphone, though we started recording on the application in the fourth session. * The abbreviation in the skill column is as follows: Expert: E, Advanced: A, Intermediate: I, Novice: N. ^+^ Time duration is in minutes.

Session	Where	When	Subjects	Skill ^*^	Techniques	Self-Recorded	Glacier	Duration ^+^
1	Hintertux	June 2019	4	AAII	3	No	Yes	50
2	Dachstein	November 2019	1	N	5	No	Yes	28
3	Galterbergalm	February 2020	1	E	6	No	No	151
4	Hintertux	July 2020	1	E	6	Partially	Yes	71
5	Ramsau	February 2021	4	EEAN	6	Yes	No	728

**Table 2:** Results for algorithm examination averaged over the other settings (window size and sliding rate). Results show that KMeans_NFS and KMeans_PCA work with slightly higher accuracy, NMI, and ARI than GMM_NFS. Even though there is not a high difference in accuracy between these three models with two Ward models, one can see that NMI and ARI drop considerably. So, we chose this model for further examination.

Algorithm	FST	Accuracy [%]	NMI	ARI	Accuracy [σ]	NMI [σ]	ARI [σ]
Kmeans	NFS	96.56	0.68	0.80	1.85	0.12	0.14
Kmeans	PCA	96.53	0.68	0.80	1.87	0.12	0.14
GMM	NFS	96.02	0.67	0.79	4.99	0.12	0.14
Ward	NFS	95.49	0.64	0.75	3.17	0.17	0.16
Ward	PCA	95.12	0.62	0.73	3.13	0.18	0.17
GMM	PCA	92.88	0.58	0.67	3.39	0.12	0.11
Baseline	–	88.7	0.0	0.0	0.45	0.0	0.0

**Table 3:** Results of Windowing strategy examination averaged over the other settings (Algorithm and FST). The experiment on window size and sliding rate shows that a 6-s window without overlap works slightly better than others, considering higher accuracy and ARI. However, all the results in this table are pretty similar.

Window Size [s]	Sliding Rate	Accuracy [%]	NMI	ARI	Accuracy [σ]	NMI [σ]	ARI [σ]
6	1	96.07	0.68	0.79	2.81	0.12	0.12
7	0.2	95.81	0.68	0.78	2.84	0.12	0.12
9	0.2	95.75	0.68	0.78	2.89	0.12	0.13
6	0.5	95.76	0.67	0.78	3.41	0.17	0.16
8	0.2	95.53	0.67	0.78	3.39	0.13	0.12

**Table 4:** Finding the best setting. The top five results show that our chosen setting (KMeans_NFS with a 6-s window and no overlap) works similarly to the highest accuracy. Additionally, the KMeans algorithm and window size of 6-s show up the most in the best results.

Algorithm	FST	Window Size [s]	Sliding Rate	Accuracy [%]	NMI	ARI	Accuracy [σ]	NMI [σ]	ARI [σ]
GMM	NFS	6	0.5	97.43	0.74	0.85	1.33	0.08	0.11
KMeans	PCA	6	0.5	97.41	0.73	0.85	1.32	0.08	0.11
KMeans	NFS	9	0.2	97.01	0.73	0.84	1.41	0.07	0.10
KMeans	NFS	6	0.5	97.32	0.72	0.84	1.26	0.07	0.10
KMeans	NFS	6	1	97.14	0.72	0.84	1.10	0.05	0.07

**Table 5:** Finding the best setting. Results show that our chosen setting (KMeans_NFS, window size of 6 s, and 100% sliding rate) works similarly to the highest accuracy. However, the table of the top five best models shows that KMeans_PCA with a 6-s window and 50% overlap performs the best due to its highest accuracy. The average baseline for this session is 91.46%.

Algorithm	FST	Window Size [s]	Sliding Rate	Accuracy [%]	NMI	ARI	Accuracy [σ]	NMI [σ]	ARI [σ]
KMeans	NFS	6	0.2	99.28	0.87	0.95	0.09	0.01	0.01
KMeans	PCA	6	0.2	99.27	0.87	0.95	0.09	0.01	0.01
KMeans	PCA	7	0.2	99.25	0.87	0.94	0.12	0.02	0.01
KMeans	PCA	8	0.5	99.25	0.87	0.94	0.10	0.02	0.01
KMeans	NFS	7	0.2	99.23	0.87	0.94	0.13	0.02	0.01

**Table 6:** Accuracy does not drop considerably due to over/under-fitting which is expected because of high baseline accuracy. In contrast, clustering metrics drop significantly as a result of under/over-fitting as they qualify the goodness of cluster analysis.

Algorithm	FST	Window Size [s]	Sliding Rate	Accuracy [%]	NMI	ARI	Detected Activities
KMeans	PCA	8	0.5	99.17	0.86	0.94	7
GMM	PCA	8	0.5	98.12	0.77	0.87	8
Baseline	-	-	-	90.76	0	0	0

## Data Availability

Not applicable.
